# Component Interaction of ESCRT Complexes Is Essential for Endocytosis-Dependent Growth, Reproduction, DON Production and Full Virulence in *Fusarium graminearum*

**DOI:** 10.3389/fmicb.2019.00180

**Published:** 2019-02-12

**Authors:** Qiurong Xie, Ahai Chen, Yunzhi Zhang, Mingyue Yuan, Wei Xie, Chengkang Zhang, Wenhui Zheng, Zonghua Wang, Guangpu Li, Jie Zhou

**Affiliations:** ^1^Key Laboratory of Biopesticide and Chemical Biology of Education Ministry, College of Plant Protection, Fujian Agriculture and Forestry University, Fuzhou, China; ^2^Fujian University Key Laboratory for Plant-Microbe Interaction, School of Life Sciences, Fujian Agriculture and Forestry University, Fuzhou, China; ^3^Institute of Oceanography, Minjiang University, Fuzhou, China; ^4^Department of Biochemistry and Molecular Biology, University of Oklahoma Health Sciences Center, Oklahoma City, OK, United States; ^5^Peggy and Charles Stephenson Cancer Center, University of Oklahoma Health Sciences Center, Oklahoma City, OK, United States

**Keywords:** *Fusarium graminearum*, ESCRT complexes, pathogenicity, interactome, endocytosis

## Abstract

Multivesicular bodies (MVBs) are critical intermediates in the trafficking of ubiquitinated endocytosed surface proteins to the lysosome/vacuole for destruction. Recognizing and packaging ubiquitin modified cargoes to the MVB pathway require ESCRT (Endosomal sorting complexes required for transport) machinery, which consists of four core subcomplexes, ESCRT-0, ESCRT-I, ESCRT-II, and ESCRT-III. *Fusarium graminearum* is an important plant pathogen that causes head blight of major cereal crops. Our previous results showed that ESCRT-0 is essential for fungal development and pathogenicity in *Fusarium graminearum*. We then, in this study, systemically studied the protein-protein interactions within *F. graminearum* ESCRT-I, -II or -III complex, as well as between ESCRT-0 and ESCRT-I, ESCRT-I and ESCRT-II, and ESCRT-II and ESCRT-III complexes and found that loss of any ESCRT component resulted in abnormal function in endocytosis. In addition, ESCRT deletion mutants displayed severe defects in growth, deoxynivalenol (DON) production, virulence, sexual, and asexual reproduction. Importantly genetic complementation with corresponding ESCRT genes fully rescued all these defective phenotypes, indicating the essential role of ESCRT machinery in fungal development and plant infection in *F. graminearum*. Taken together, the protein-protein interactome and biological functions of the ESCRT machinery is first profoundly characterized in *F. graminearum*, providing a foundation for further exploration of ESCRT machinery in filamentous fungi.

## Introduction

The filamentous fungus *Fusarium graminearum* is the major causal agent of Fusarium head blight (FHB) disease of cereal crops including wheat, barley, and other small grains (Goswami and Kistler, 2004; Starkey et al., 2007). Epidemics of this destructive disease can destroy high-yielding crop within a few weeks and result in significant yield losses (Mcmullen et al., 1997). Additionally, *F. graminearum* can produce mycotoxins such as deoxynivalenol (DON) and zearalenone in infected crops, which are hazardous to humans and animals (Mcmullen et al., 1997; Desjardins, 2003; Pestka and Smolinski, 2005). To date, effective fungicides for managing FHB are not available yet. Therefore, a better understanding of regulation mechanisms associated with fungal development, FHB pathogenesis, and DON biosynthesis will be necessary to facilitate the development of efficient control strategies against this devastating pathogen.

The ESCRT (Endosomal Sorting Complexes Required for Transport) machinery was discovered in *Saccharomyces cerevisiae* by Emr and colleagues for sorting ubiquitinated membrane proteins into the lumen of the lysosome-like vacuole for degradation (Katzmann et al., 2001; Babst et al., 2002a,b). In yeast, the machinery consists of four distinct protein subcomplexes, ESCRT-0, ESCRT-I, ESCRT-II, ESCRT-III, plus several accessory proteins (Hurley, 2008, 2015). ESCRT-0 complex (including Vps27 and Hse1 components) initiates the ESCRT pathway. The FYVE (named after Fab1, YOTB, Vac1 and EEA1) zinc finger domain of Vps27 binds to the endosomal specific lipid phosphatidylinositol 3-phosphate (PtdIns3P), targeting the entire ESCRT-0 complex to endosomes (Gaullier et al., 1998; Katzmann et al., 2003). Ubiquitinylated (Ub-) cargoes were bound and clustered on endosomes via interaction with the two ubiquitin interacting motifs (UIMs) on Vps27 (Bilodeau et al., 2002, 2003). In addition to the UIM motifs, both Vps27 and Hse1 subunits contain a N-terminal VHS domain, which has been shown to cooperate in high-avidity binding to polyubiquitinated cargoes (Ren and Hurley, 2010). The soluble hetero-tetramer ESCRT-I complex, consisting of Vps23, Vps28, Vps37, and Mvb12 (Katzmann et al., 2001; Chu et al., 2006; Curtiss et al., 2007), is recruited to the endosomal menbrane via the interaction between the N-terminal UEV (Ubiquitin E2 Variant) domain of its Vps23 subunit and P(S/T)XP motifs of Vps27 (Katzmann et al., 2003; Kostelansky et al., 2006). In addition to ESCRT-0, the UEV domain also interacts with ubiquitinated proteins (Katzmann et al., 2001; Teo et al., 2004) and hands off the cargoes to ESCRT-II complex, which includes three subunits, Vps22, Vps36, and Vps25 (Babst et al., 2002b). Like the FYVE domain of upstream MVB sorting component Vps27, GLUE (GRAM-like ubiquitin-binding in EAP45) domain of Vps36 subunit provides endosomal localization by binding preferentially to PtdIns3P (Teo et al., 2006). Two NpI4 type zinc fingers, NZF-C, and NZF-N, are inserted into the GLUE domain. The NZF-N domain is required for binding to the C-terminal domain of Vps28 and thereby interacts with the ESCRT-I complex (Gill et al., 2007), while the other NZF domain recognizes the monoubiquitylated proteins (Alam et al., 2004). Membrane-bound ESCRT-II complex recruits the downstream ESCRT-III complex through the interaction between Vps25 and Vps20, a subunit of the ESCRT-III complex. In contrast to other ESCRT complexes (ESCRT-0, ESCRT-I, and ESCRT-II), the four core subunits of ESCRT-III (Vps20, Snf7, Vps24, and Vps2) does not form a stable complex. ESCRT-III subunits exist in the cytosol as monomers and only transiently assemble into active complex on endosomal membrane (Babst et al., 2002a). In addition to the four core subunits, ESCRT-III complex also contains several accessory proteins, including Did2 (Doa-4 independent degradation-2), Ist1 (increased salt tolerance-1), Bro1/Alix (BCK1-like resistance to osmotic shock protein-1/apoptosis-linked gene-2 interacting protein X), and Vps60 (Nickerson et al., 2006; Dimaano et al., 2008; Rue et al., 2008). To complete ESCRT cycle and replenish the cytosolic pool of ESCRT-III subunits, Vps4, the type I AAA-ATPase, is recruited to the ESCRT-III complex by binding to Vps2 and provides energy needed for removal of ESCRT-III complex from endosomal membrane through ATP hydrolysis (Babst et al., 1997, 1998). Vta1, another component of Vps4 complex, binds to Vps4 and enhances ATPase activity and ESCRT-III binding (Yeo et al., 2003; Shiflett et al., 2004; Lottridge et al., 2006; Shestakova et al., 2010).

In addition to yeast, the ESCRT machinery is conserved in higher mammalian systems and plays a similar role in sorting ubiquitinated membrane proteins into lysosomes for degradation (Hurley, 2010). However, the mammalian ESCRT machinery is more complex than its yeast counterpart as multiple isoforms of several mammalian ESCRT subunits exist (Hurley, 2015). There is increasing awareness that dysfunction of ESCRT components is associated with various human diseases (Saksena and Emr, 2009; Stuffers et al., 2009). The ESCRT machinery has been shown to play an important role in tumor suppression. The expression of Vps37A/HCRP1 (hepatocellular carcinomas-related protein 1), named for its inhibitory role in proliferation and invasion of hepatocellular carcinomas cell lines, is dramatically reduced in hepatocellular carcinomas (Xu et al., 2003). Mutations in CHMP2B (Vps2 homolog) were identified in some patients with FTD (frontotemporal dementia) and in ALS (amyotrophic lateral sclerosis) (Skibinski et al., 2005; Parkinson et al., 2006). In addition, Tsg101 (Vps23 homolog) was shown to be associated with the release of HIV (Pornillos et al., 2002).

In plants, there are ESCRT-I, ESCRT-II, and ESCRT-III complexes but not ESCRT-0, suggesting a relatively conserved role of the ESCRT machinery among eukaryotes. In addition to Vps27/Hrs (mammalian homolog of yeast Vps27) and Hse1/STAM (mammalian homolog of yeast Hse1), two other protein families, GGAs (Golgi-localized, γ-ear-containing, ADP-ribosylation-factor-binding protein) and TOM1 (Target of Myb1) also contain VHS domain that binds and recruits ubiquitinated cargoes on endosomal membranes (Puertollano and Bonifacino, 2004; Puertollano, 2005). Interestingly, whereas no GGAs have been found in plants, *Arabidopsis* genome contains nine TOL (TOM1-LIKE) genes (Winter and Hauser, 2006), which may be the functional equivalent of ESCRT-0 and provide an alternative mechanism for recognizing and binding ubiquitinated cargoes (Blanc et al., 2009). Many studies have also shown that endosomal sorting mediated by ESCRT machinery contributes to diverse physiological processes in plants (Fan et al., 2015; Paez et al., 2016). Mutations in *Arabidopsis* ESCRT-I subunits Vps28-2 and Vps37-1 do not have any effect on plant development, but lead to defective internalization of FLS2 (FLAGELLIN-SENSING 2) (Spallek et al., 2013), which can recognize the flagellin or the flagellin peptide derivative flg22 and activate defense immunity after endocytosis (Robatzek et al., 2006). The loss of rice Vps22, a major component of ESCRT-II complex, leads to seedling lethality and growth defects of root and shoot (Zhang et al., 2013). The inducible overexpression of ESCRT-III dominant negative mutants leads to severe cellular and developmental defects, including loss of the central vacuole, reduced cell size and abnormal chloroplast development in mesophyll cells (Cai et al., 2014).

All of the above studies showed that a better understanding of ESCRT biology is of genetic, biomedical and ecological importance. However, little is known about the function of ESCRT machinery in filamentous fungi. ESCRT core complexes including ESCRT-0, -I, -II, and -III can be sequentially recruited and assembled on the endosomal membrane during MVB formation in yeast, plant, and human cells by multiple interactions among these complexes (Saksena et al., 2007; Otegui and Spitzer, 2008; Hurley, 2010, 2015; Henne et al., 2011). Several studies have identified these interactions of ESCRT complexes from different organisms by taking advantage of yeast two-hybrid assays (Martin-serrano et al., 2003; Von Schwedler et al., 2003; Bowers et al., 2004; Richardson et al., 2011). However, the interaction network of ESCRT core complexes and working model of ESCRT assembly in filamentous fungi have not been established. In this study, we therefore employed the yeast two-hybrid system to identify the multitude of protein–protein interactions that occur within and between the ESCRT core complexes in *F. graminearum*. Furthermore, we also continued to characterize the biological functions of the ESCRT machinery based on our first exploration of ESCRT-0 function in *F. graminearum* (Xie et al., 2016). Our work will serve as a framework for testing ESCRT assembly and function(s) in other filamentous fungi, and systematically understanding the function of the ESCRT machinery.

## Materials and Methods

### Strains, Culture Conditions

The PH-1 strain was used as the wild-type strain in this study. Culture conditions were the same as described previously using complete medium (Zheng et al., 2012; Chen et al., 2016). Sensitivities to environmental stimuli were examined on complete medium supplemented with 1 mg/ml CR, 200 mg/ml Calcofluor white (CFW), 0.01% SDS, 1M NaCl, 1M KCl, and 10 mM H_2_O_2_.

### Disruption of ESCRT Genes in *F. graminearum*

The *F. graminearum* ESCRT genes were disrupted by gene replacement. To replace the *FgVPS23*, the 1,056-bp upstream and 816-bp downstream fragments of *FgVPS23* were amplified with primer pairs 1F/1R and 2F/2R (Supplementary Table 2), respectively. And then Splicing Overlap Extension (SOE)-PCR was used to connect the resulting amplicons to the hph fragments (Catlett et al., 2003). The resulting PCR fragments containing the gene replacement cassette were transformed into protoplasts of wild-type strain PH-1 as described (Hou et al., 2002). After transformation, transformants with hygromycin-resistance were picked individually and PCR analyses with designated primer pairs 3F/3R and 4F/4R were performed to identify transformants that carried the insertion of *hph* at the *VPS23* locus. Southern blot analysis was performed according to standard manufacturer's protocols (Digoxigenin High Prime DNA Labeling and Detection Starter Kit I; Roche) to further confirmed the mutant strains. Similar method was used to generate other ESCRT genes deletion mutants.

### Construction of GFP Fusion Vectors and Transformation

To generate FgVps23-GFP fusion vector, the *FgVPS23* gene with its 1515-bp upstream promoter region was amplified with 29F and 29R using genomic DNA extracted from wild type (PH-1) as template. The resulting fragment was cloned into the *Nde* I and *Eco*R I sites of the PKNTG vector harboring the GFP allele and the neomycin gene as a selection marker via using the ClonExpress One Step Cloning Kit (Vazyme, Nanjing, China). Other ESCRT protein GFP fusion vectors were generated by using the same strategy. The resulting vector was verified by sequencing and then transformed into the protoplasts of corresponding ESCRT gene deletion mutant strains. The neomycin-resistant transformants were picked and then screened by PCR and GFP signal.

### Asexual and Sexual Reproduction Assays

For asexual reproduction assays, mycelial agar blocks (5 mm in diameter) were inoculated into CMC liquid medium containing 0.1% NH_4_NO_3_, 0.1% yeast extract, 0.1%KH_2_PO_4_, 0.05% ${\text{MgSO}}_{4}^{*}$7H_2_O, 1.5% carboxylmethyl cellulose or synthetic low-nutrient agar (SNA) medium containing 0.1% KNO_3_, 0.05% MgSO_4_·7H_2_O, 0.02% glucose, 0.1% KH_2_PO_4_, 0.05% KCl, 0.02% sucrose, and 2% agar. The number of conidia were determined 6 days after incubation at 25°C under microscopy by using a hemacytometer. Since *F. graminearum* is homothallic, to induce sexual reproduction, mycelial agar blocks were placed on carrot agar and incubated at 28°C for a week. Then aerial hyphae were removed and the plates were gentle pressed down with 300μl of sterile 0.1% Tween 20. All of the sexually-induced cultures were incubated at 28°C for an additional 2–3 weeks under a 12 h dark/12 h black light cycle. Each experiment was repeated independently three times.

### Virulence Test and DON Assay

The virulence of fungal strains was determined on flowering wheat heads. Mycelial agar blocks (3 mm in diameter) of wild-type strain, mutant strains and the corresponding complementation strains were incubated at the middle spikelet of wheat. The inoculated wheat heads were enclosed in small plastic bags misted with water for 3 days to maintain the moisture. After incubation for 14 days, spikelets with typical head blight symptoms were examined and counted to estimate the disease index. All the infection assays were repeated at least three times. For determination of DON production, tested strains were cultured in liquid trichothecene biosynthesis induction (TBI) medium (Gardiner et al., 2009) in the dark for 1 week and the amount of DON was measured by using DON detection plate kit (Finder Biotech Co. Ltd, China).

### Yeast Two-Hybrid Assay

The yeast two-hybrid assay was used to examine the possible protein-protein interactions among ESCRT proteins in accordance with the manufacturer's protocol (Matchmaker GAL4 Two-Hybrid System 3; Clontech). To generate vectors for yeast two-hybrid analyses, the full-length cDNA of each tested gene was amplified from first-strand cDNA of PH-1 with primers listed in Supplementary Table 2. The cDNA of each gene was cloned into the yeast GAL4 activation domain vector pGADT7 and GAL4-binding domain vector pGBKT7 as the prey vector and bait vector, respectively. The resulting bait and prey vectors were confirmed by sequencing and were co-transformed in pairs into the *S. cerevisiae* reporter strain AH109 (Clontech) via the lithium acetate transformation procedure (Schiestl and Gietz, 1989). The isolation and confirmation of transformants were conducted as described (Chen et al., 2008). Briefly, the Leu+ and Trp+ transformants were isolated and assayed for growth on SD-Ade-Leu-Trp-His with the addition of 40 μM X-gal to examine the *HIS3* reporter gene expression and β-galactosidase activity. In all assays, yeast transformants expressing the pGBKT7-P53 bait-pGADT7-T prey and pGBKT7-Lam bait-pGADT7-T prey constructs were used as the positive control and negative control, respectively. All the yeast two-hybrid assays were performed three times to confirm the results. All primers used in this experiment were listed in Supplementary Table 2.

### Quantitative RT-PCR Analysis

For quantitative RT-PCR (qRT-PCR) assays, RNA isolation was performed with the RNA extraction kit following the instructions provided by the manufacturer. First-strand cDNA was generated using the PrimeScript^TM^ RT reagent kit (Takara). The resultant cDNA was used as a template for qRT-PCR. qRT-PCR was performed with SYBR Premix Ex Taq^TM^ (Takara), denaturation at 95°C, 32 s of annealing at 60°C for 40 cycles. The relative abundance of the transcripts of each gene were calculated by 2^−ΔΔ*T*^ (Livak and Schmittgen, 2001) using *F. graminearum* tublin gene (*FGSG_09530*) as control. Primers used to amplify selected genes in qRT-PCR reactions are listed in Supplementary Table 2.

## Results

### Interaction Networks of ESCRT Core Complexes in *F. graminearum*

**Figure 1:**
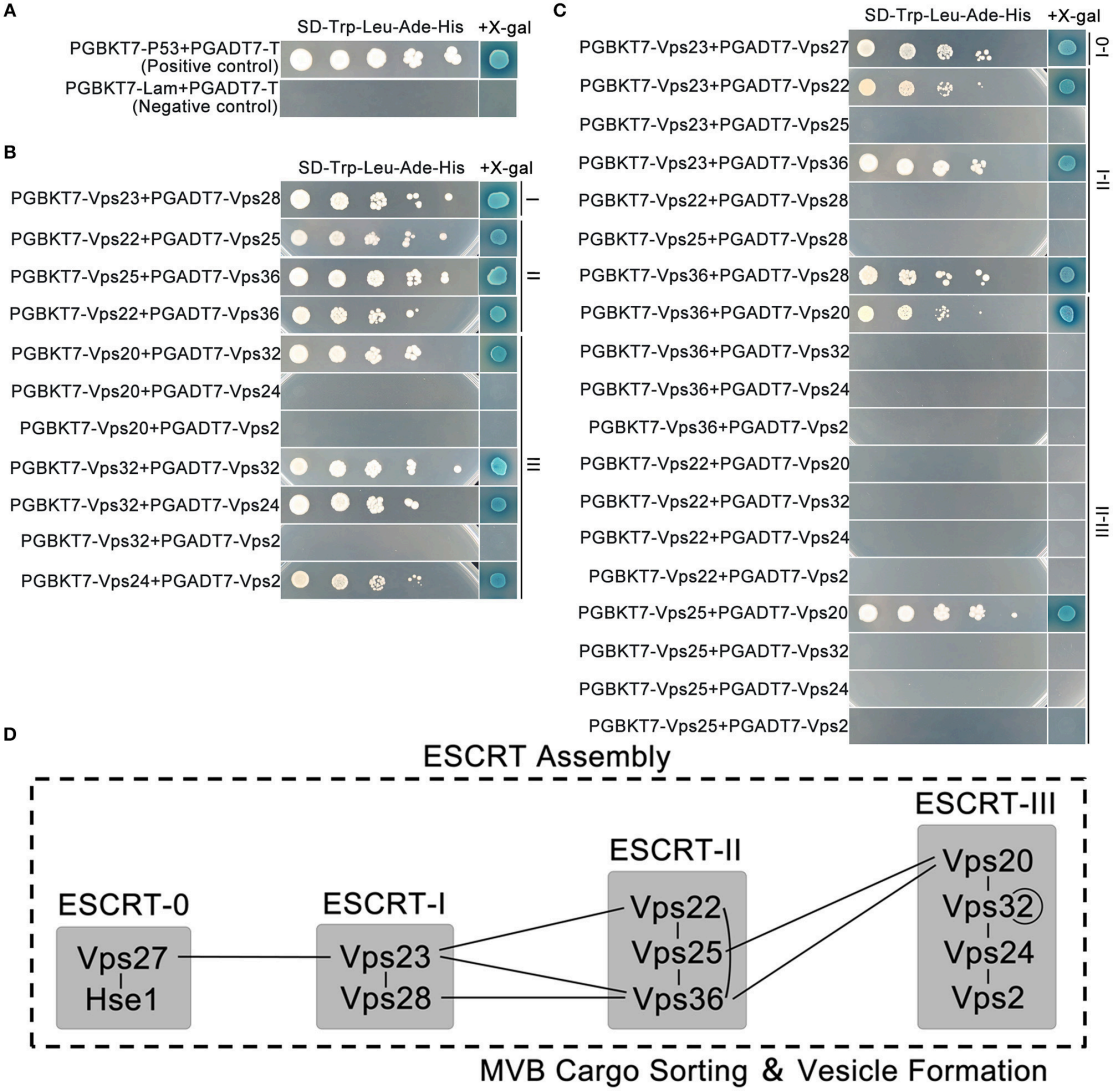
Interactions of the ESCRT machinery based on the yeast two-hybrid assay. **(A)** The interaction of pGBKT7-53/pGADT7-T and pGBKT7-Lam/pGADT7-T were used as the positive and negative control, respectively. Interactions within **(B)** and between **(C)** ESCRT subcomplexes. Yeast transformants carrying the indicated constructs were diluted into different concentrations and plated onto the selective plates supplemented with X-gal and without Leu/Trp/His/Ade for assaying the growth and β-galactosidase (LacZ) activities. **(D)** A model depicting the protein-protein interactions of ESCRT machinery. Lines indicate the interactions that we have showed in this study.

Previously, we identified the interactions within the ESCRT-0 complex, and showed that its two components FgVps27 (*FGSG_08545*) and FgHse1 (*FGSG _08492*) could directly interact with each other (Xie et al., 2016). In this study, we focused on protein-protein interactions within *F. graminearum* ESCRT-I, -II, and –III complexes, as well as between these complexes (Supplementary Table 1). To identify orthologs of components of ESCRT-I, -II, and -III complexes in *F. graminearum*, the sequences of corresponding ESCRT genes from the budding yeast *S. cerevisiae* were used for BLASTP searches in the *F. graminearum* genome database (http://www.broadinstitute.org/annotation/genome/fusarium_group/MultiHome.html). In total, 9 genes *FGSG_02656, FGSG_000291, FGSG_04120, FGSG_08401, FGSG_05263, FGSG_04112, FGSG_10883, FGSG_10832, FGSG_10092* were found. They are renamed according to their orthologs in *S. cerevisiae* (Supplementary Figure 1). However, there are no Vps37 and Mvb12 in *F. graminearum*, suggesting *F. graminearum* may have lost the Vps37 and Mvb12 orthologs during evolution. To determine the interactions between ESCRT core components, yeast two-hybrid was performed between ESCRT components within or between complexes I, II, and III. With proper positive and negative controls (Figure 1A), we found strong interactions between individual components with high β-galactosidase signal within ESCRT-I and ESCRT-II complexes, while ESCRT-III complex also displayed several interactions, including FgVps20-FgVps32, FgVps32-FgVps24, and FgVps24-FgVps2 in addition to FgVps32 self-interaction (Figure 1B). Between the ESCRT complexes, the interaction of FgVps27 and FgVps23 connected ESCRT-0 and ESCRT-I complexes whereas the interactions of FgVps23-FgVps22, FgVps23-FgVps36, and FgVps28-FgVps36 facilitated the association of ESCRT-I and ESCRT-II. FgVps20 interacted with FgVps25 and FgVps36, linking ESCRT-II and ESCRT-III complexes (Figure 1C). Taken together, our results established the interactome of ESCRT core complexes in *F. graminearum* (Figure 1D).

### Generation of ESCRT Gene Deletion Mutants

In order to investigate the biological functions of ESCRT genes in *F. graminearum*, we attempted to generate gene deletion mutants for every ESCRT component of ESCRT-I, -II, and -III complexes by replacing each gene with a selective marker [the bacterial phosphotransferase (*hph*) gene], through transformation of protoplasts of the wild-type *F. graminearum* strain with the deletion constructs. The resulting hygromycin-resistant transformants of each ESCRT gene were identified by PCR analyses with gene-specific primers listed in Supplementary Table 2. For 6 genes (*FgVPS23, FgVPS28, FgVPS22, FgVPS25, FgVPS36, FgVPS20*), at least two independent deletion mutants were identified for each gene with similar phenotypes as described below, and further confirmed by Southern hybridization assays (Supplementary Figure 2). For the other 3 genes (*FgVPS2, FgVPS24, FgVPS32*), which belonged to the ESCRT-III complex, we failed to obtain gene knockout mutants after screening thousands of transformants from many independent transformation experiments, suggesting that these genes are probably essential for *F. graminearum* growth or development (Son et al., 2011; Wang et al., 2011; Yun et al., 2015). To further confirm that the defects displayed in ESCRT mutants were caused by the loss of corresponding ESCRT proteins, native ESCRT genes with their native promoters, were reintroduced into corresponding gene deletion mutants. The corresponding complementation strains were obtained after PCR and phenotypic screening and named as Δ*Fgvps23-C*, Δ*Fgvps28-C*, Δ*Fgvps22-C*, Δ*Fgvps25-C*, Δ*Fgvps36-C*, and Δ*Fgvps20-C*, respectively, in this study.

### ESCRTs Are Essential for Proper Endocytosis

**Figure 2:**
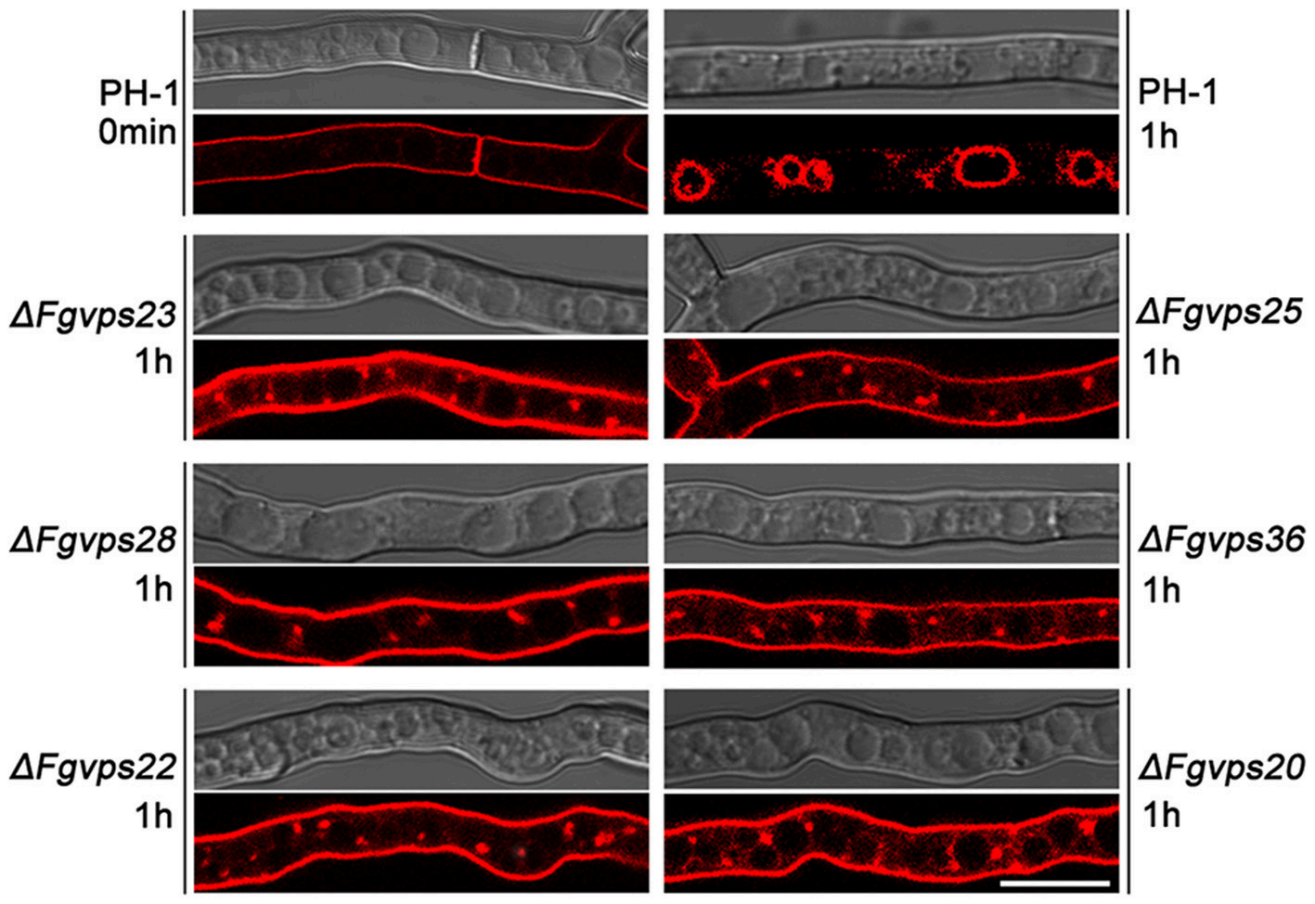
Deletion of ESCRT-I, -II, and -III components perturb the transport of FM4-64 from the plasma membrane to the vacuolar membrane. Cells of indicated strains were stained with 8 mM FM4-64 and the internalization of FM4-64 was observed under Nikon A1R laser scanning confocal fluorescence microscope. Photographs were taken at indicated periods. Bar = 10 μm.

Endocytosis constitutes a fundamental eukaryotic function that internalizes fluids, solutes, and plasma membrane components into vesicles which incorporate with the endosomal system (Huotari and Helenius, 2011). Our previous study showed that FgVps27, the core component of ESCRT-0 complexes, was involved in endocytosis of *F. graminearum* (Xie et al., 2016). To determine whether other ESCRT components have a similar function, we followed the uptake of FM4-64, a lipophilic dye endocytosed and trafficked to the vacuolar membrane (Vida and Emr, 1995; Fischer-parton et al., 2000), to monitor endocytosis kinetics in the mycelial cells of wild-type and ESCRT gene mutants. As shown in Figure 2, upon FM4-64 application, the plasma membrane and septum were immediately stained in wild-type strain PH-1, and after 1 h incubation, this fluorescent dye was delivered to the vacuolar membrane by normal endocytosis. Similar staining pattern of FM4-64 was observed in ESCRT gene deletion mutants initially (data not shown). However, FM4-64 failed to be transported and targeted to vacuolar membranes, and instead, predominantly appeared in the plasma membrane and small punctate compartments adjacent to the vacuole in all the mutants after incubation for 1 h (Figure 2). Even for 2 h, ESCRT gene deletion mutants failed to deliver FM4-64 to the vacuolar membrane (data not shown), indicating that defects in proper endocytosis caused by the loss of ESCRT genes were not time-dependent. These results suggest that similar to ESCRT-0, ESCRT-I, -II, and -III complexes play critical roles in endocytic trafficking to the vacuole in *F. graminearum*.

### ESCRTs Are Required for Hyphae Development and Stress Responses

**Figure 3:**
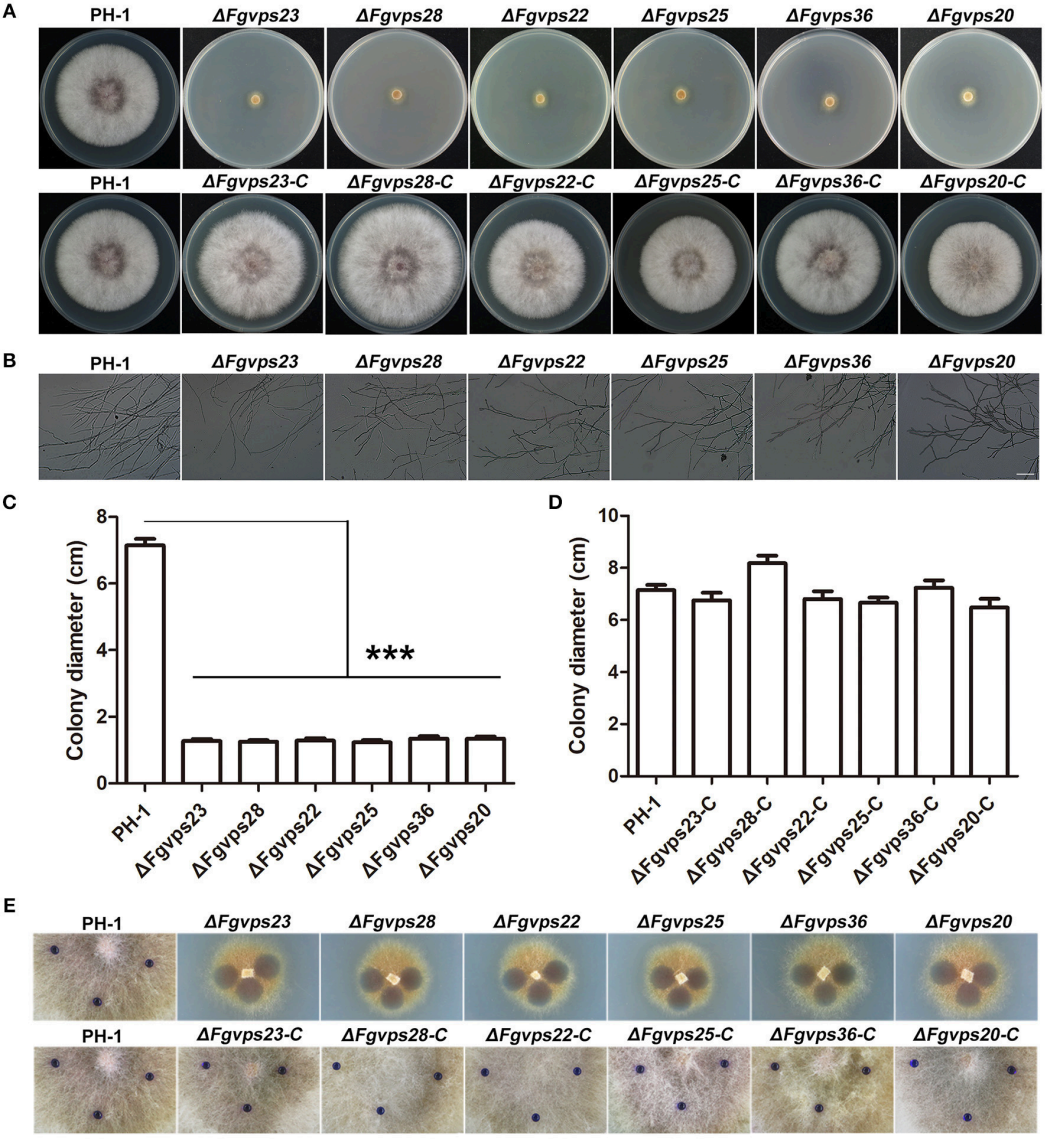
The loss of ESCRT-I, -II, and -III components cause a significant reduction in growth and hydrophobicity of aerial hyphae. **(A)** Colony of the wild-type strain PH-1, ESCRT gene deletion mutants and corresponding complementary strains grown on complete medium. **(B)** Microscopy images of mycelial morphology and branching patterns of PH-1 and ESCRT gene deletion mutant strains on CM agar. Bar = 100 μm. **(C,D)** Colony diameters of indicated strains were measured after incubation at 25°C for 3 days. Line bars in each column represent the standard deviation (SD) from three independent experiments. ^***^p < 0.001; Student's *T* test was used. **(E)** Hydrophobic character of the wild-type strain PH-1, ESCRT gene deletion mutants and corresponding complementary strains, measured 15 s after deposition of the 2.5% bromophenol blue droplets.

Endocytosis plays an essential role in cellular responses to environmental stimuli, which is critical for cell survival and proper development (Murphy et al., 2005; Fan et al., 2015; Paez et al., 2016). The deletion of ESCRT genes resulted in defective endocytosis in *F. graminearum*, suggesting that development and differentiation of *F. graminearum* may also be affected in the mutants. To test this, wild-type strain, ESCRT deletion mutants, together with corresponding complementation strains, were cultured on complete medium and incubated at 25°C for 3 days. As shown in Figure 3A, the colonies of mutants were yellowish whereas those of the wild type strain, and complemented strains were pinkish, suggesting that ESCRT may be involved in red pigment formation. In addition, the loss of ESCRT components led to severe defects in vegetative growth, displaying smaller colony morphology and thinner aerial hyphae in comparison to the wild-type (Figures 3A,C,D). Microscopic examination further showed that deletion of ESCRT components resulted in more sparse aerial hyphae than PH-1 (Figure 3B). Previous studies showed that the surface hydrophobicity of aerial hyphae is closely related to hyphal formation in many fungal species (Kershaw and Talbot, 1998; Wösten et al., 1999), suggesting that the mutants with fewer aerial hyphae may have reduced hyphal hydrophobicity. To test this contention, 2.5% bromophenol blue droplets were added onto the colony surface of each strain and observed for their absorption and dispersion. While the wild-type strain and complemented strains exhibited strong hydrophobicity and the bromophenol blue droplets didn't disperse within 30 min, the droplets placed on the colony surface of all the ESCRT mutants dispersed immediately (Figure 3E), suggesting that the deletion of ESCRT genes resulted in the loss of hydrophobicity on the mycelia surface. Taken these results together, the depletion of ESCRT impairs the hyphae development of *F. graminearum*, possibly due to the defects in endocytosis.

**Figure 4:**
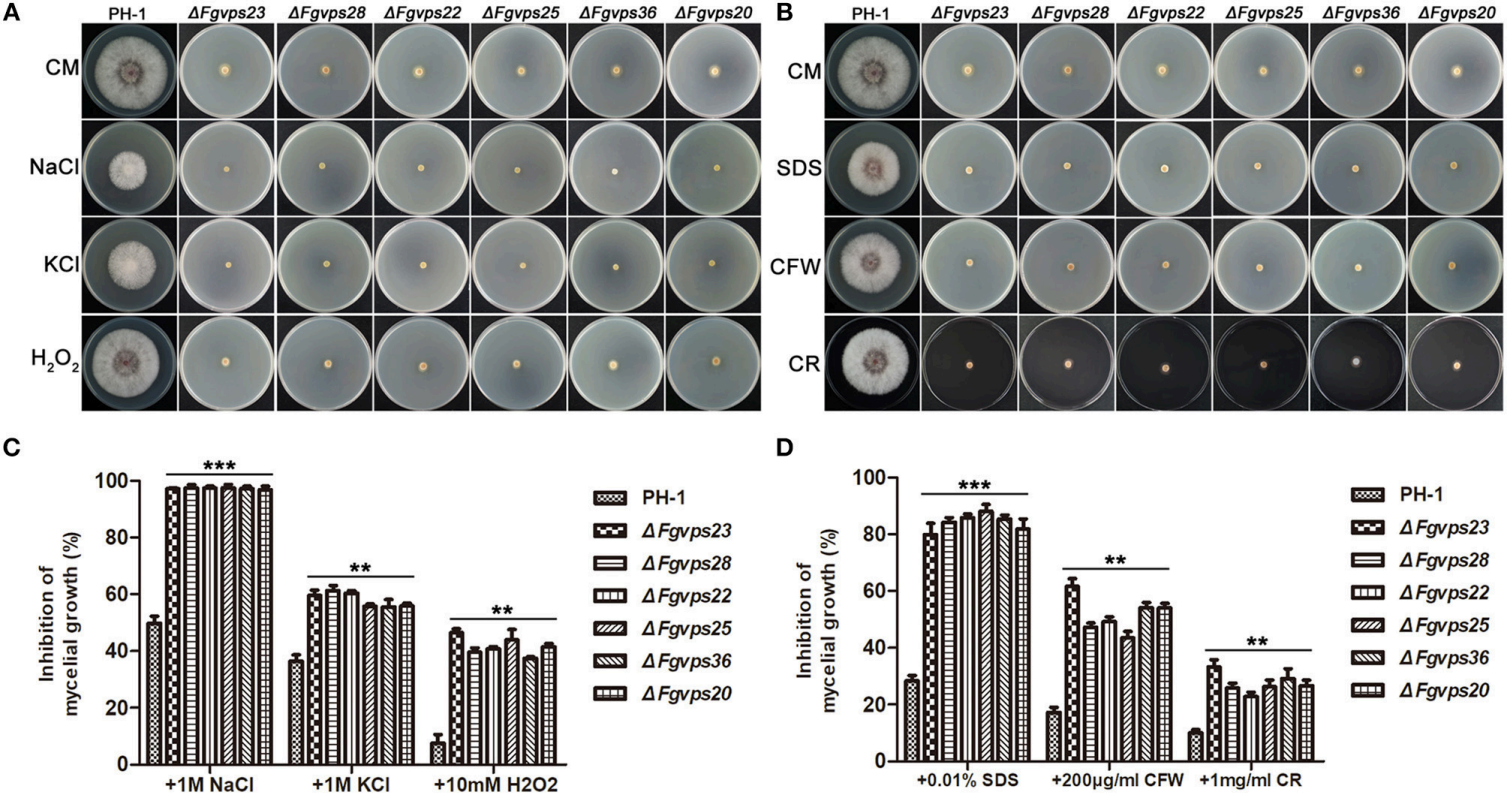
Defects of the ESCRT-I, -II and -III gene deletion mutants in response to various stressors. **(A)** The wild-type strain PH-1 and ESCRT gene deletion mutants were cultured on complete medium with hyperosmotic and oxidative stressors. **(B)** The indicated strains were cultured on complete medium supplemented with cell wall and cell membrane damaging agents. **(C,D)** The growth inhibition rate of the indicated strains under different stress conditions. Line bars in each column represent the standard deviation (SD) from three independent experiments. ^**^p < 0.01; ^***^p < 0.001; Student's *T* test was used.

Next, we investigated whether the dysfunction in endocytic pathway due to the loss of ESCRT genes also had an effect on response to various environmental stresses in *F. graminearum*. When strains were cultured on CM plates with 1M NaCl and 1M KCl for 3 days, the growth of these ESCRT mutants was almost completely blocked in comparison to that of the wild type (Figures 4A,C), indicating that the ESCRT core components in *F. graminearum* are indispensable for regulating responses to hyperosmotic stress. Furthermore, both ESCRT mutants were hypersensitive to oxidative stress and their growth was more significantly reduced by the addition of 10 mM H_2_O_2_ (Figures 4A,C)_._ Therefore, deletion of the ESCRT core components may result in decreased expression of genes involved in ROS scavenging. We also determined sensitivity of the ESCRT mutants to a series of damaging agents of cell membrane and cell wall. As shown in Figures 4B,D, the presence of 0.01% SDS severely inhibited the growth of all mutants in comparison with that of PH-1. Likewise, the ESCRT mutants grew slower than the wild type with the treatment of Calcofluor white (CFW) and Congo Red (CR), suggesting that the ESCRT core components play an important role in the cell wall integrity (CWI) pathway in *F. graminearum*.

### Disruption of ESCRT Genes Blocks the Perithecium Production and Conidium Formation

**Figure 5:**
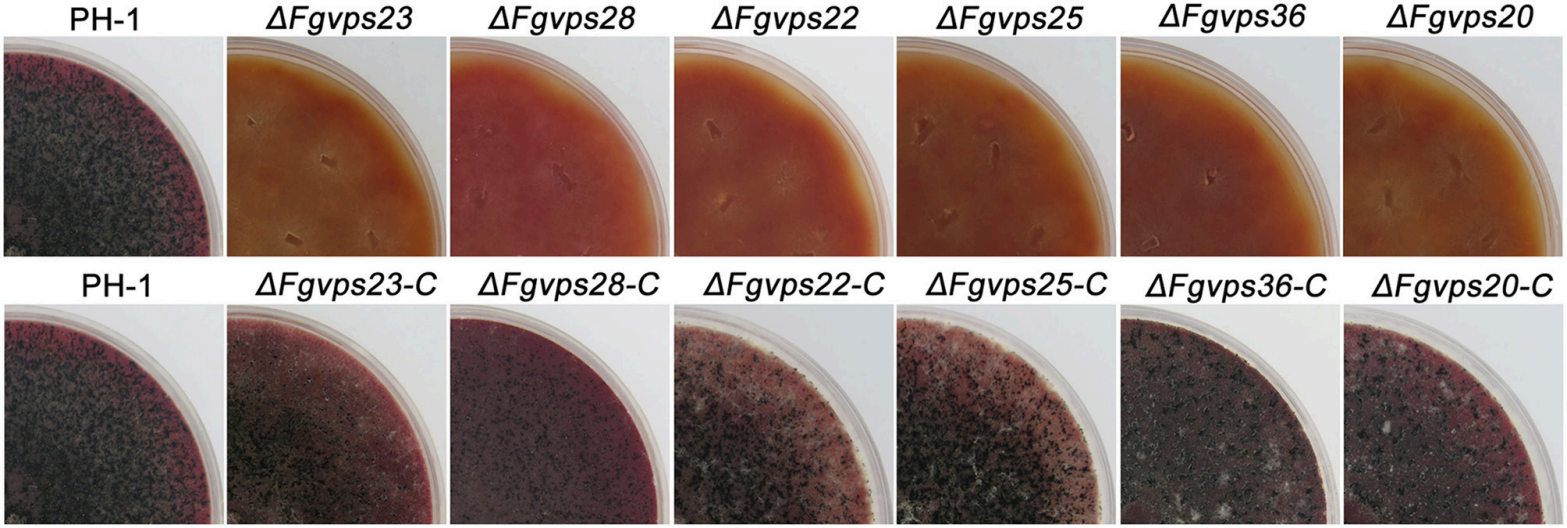
The ESCRT gene deletion mutants were defective in sexual reproduction. Mating cultures of the wild-type strain PH-1, ESCRT gene deletion mutants and corresponding complementary strains. The photographs were taken 2 weeks after sexual induction. Perithecia were only produced by PH-1 and complementary strains.

Sexual and asexual reproduction play a critical role in the infection cycle of *F. graminearum* since ascospores are the primary inoculum and conidia are required for host colonization and disease spreading (Trail et al., 2002; Dgiii et al., 2005; Yang et al., 2015). To test the role of ESCRT components in sexual development, wild-type strain, deletion mutants, and corresponding complemented strains were cultured on carrot agar plates. Abundant perithecia were found on the plates of wild-type strain and complemented strains 14 days post-fertilization. In contrast, mutants were unable to produce any perithecium under the same conditions (Figure 5). For asexual reproduction, fresh mycelial plugs taken from wild-type strain, mutants and corresponding complemented strains were inoculated into liquid carboxymethylcellulose (CMC) media (Cappellini and Peterson, 1965). After incubation for 6 days, the ESCRT mutants failed to produce any conidia while the wild type produced (127.86 ± 12.64) × 10^4^ macroconidia per milliliter. To rule out the possibility of medium dependency in conidiation, the conidiation of these strains were also investigated on synthetic low-nutrient agar (SNA) plates, and similar results were obtained. These results indicate that ESCRT components are critical for sexual and asexual development in *F. graminearum*.

### ESCRT Components Are Required for Plant Infection and DON Production

**Figure 6:**
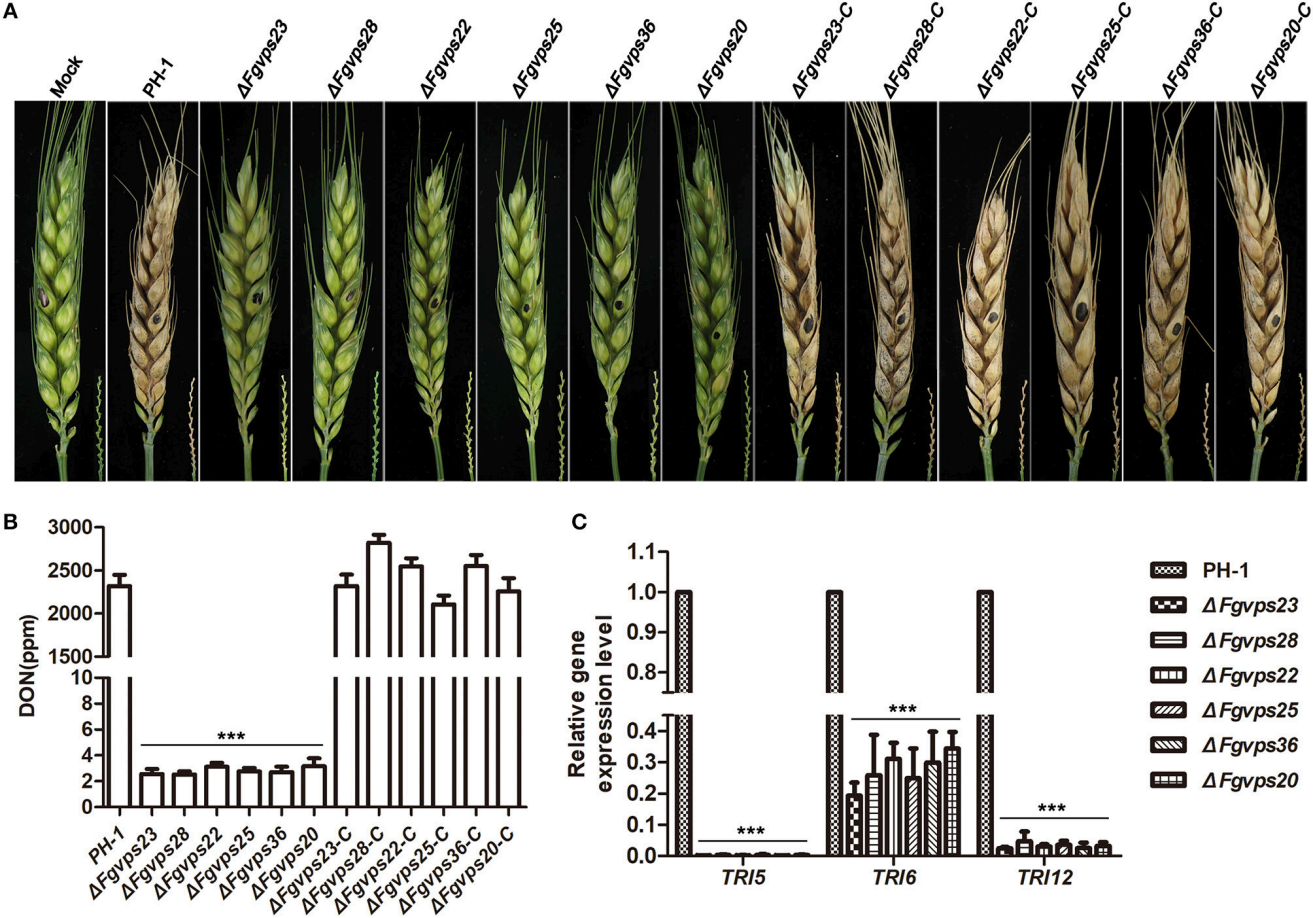
ESCRT-I, -II and -III components are required for plant infection and DON production. **(A)** Wheat heads were point inoculated with wild-type strain, ESCRT gene deletion mutants and corresponding complementary strains. Photographs were taken 14 days after inoculation. Black dots indicate the inoculated spikelet in each head. Wheat heads inoculated with water was used as the negative control. The bottom right hand corner of each wheat head shown the disease symptom of wheat head rachis. **(B)** DON production in 7-day-old TBI cultures of the wide type strain PH-1, ESCRT gene deletion mutants and corresponding complementary strains. **(C)** The relative expression level of DON synthesis-related gene *TRI5, TRI6*, and *TRI12* in wide type strain PH-1 and ESCRT gene deletion mutants. Their expression in wild-type stain was set to 1. Line bars in each column represent the standard deviation (SD) from three independent experiments. ^***^p < 0.001; Student's *T* test was used.

To determine whether ESCRT components play a role in plant infection, the virulence of all strains were evaluated on flowering wheat heads by point inoculation. As shown in Figure 6A, the wild-type strain PH-1 and the complementation strains colonized the host and spread rapidly from inoculated spikelet to other spikelets through rachis 14 days post-inoculation, resulting in typical scab symptoms in the wheat. However, mutants were unable to cause any symptom on the inoculated wheat kernel and failed to spread to adjacent rachis and neighboring spikelets, suggesting that ESCRT components are required for plant infection.

Deoxynivalenol (DON), a potent mycotoxin, has been identified as an important virulence factor produced by *F. graminearum* during plant infection (Seong et al., 2008; Hallen-Adams et al., 2011). We speculated that the impaired virulence of ESCRT mutants was partially due to the reduction in DON production. To test this contention, we measured the amount of DON production in the ESCRT mutants, in comparison with wild-type strain PH-1 and the complementary strains by using ELISA based DON detection plate kit. Consistent with significant reduction in virulence, DON production in ESCRT mutants was almost undetectable whereas more than 2,000 ppm DON was produced in the wild-type strain and the corresponding complementary strains (Figure 6B).

In addition, we also determined the transcription levels of the DON biosynthesis-related genes *TRI5* and *TRI6*, the trichothecene efflux pump gene *TRI12* by qRT-PCR. Consistently, the expression levels of these three genes, especially *TRI5* and *TRI12*, were decreased dramatically in the ESCRT mutants in comparison with that of PH-1 (Figure 6C). Taken together, ESCRT components positively regulate DON production in *F. graminearum*.

### Localization of ESCRT Components in *F. graminearum*

**Figure 7:**
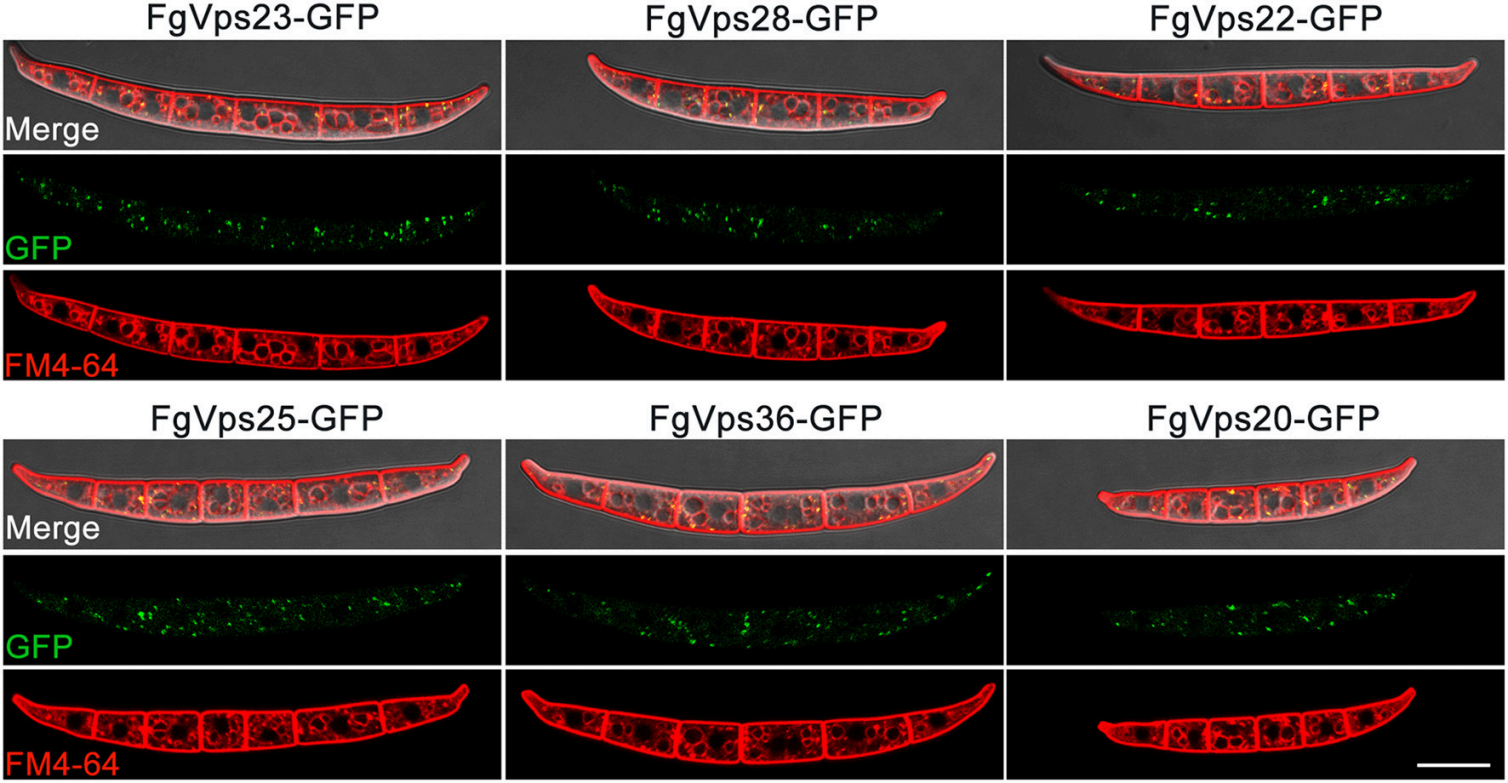
Subcellular localization of ESCRTs in *F. graminearum*. Green fluorescent protein (GFP) was fused to the C-terminus of each ESCRT gene, which was driven by native promoter. The resulting constructs were transformed into corresponding ESCRT gene deletion mutants. The conidium of indicated strains were stained with 8 mM FM4-64 followed by imaging with a Nikon A1R laser scanning confocal fluorescence microscope. GFP, FM46-4, and differential interference contrast (DIC), GFP and Fm4-64 overlay images of the same field are shown. Bar = 20 μm.

In *Aspergillus nidulans*, Vps23 had been reported to localized in endosomes (Calcagno-Pizarelli et al., 2011; Galindo et al., 2012). Moreover, our previous study showed that ESCRT-0 component FgVps27 was localized to punctate structures adjacent to the vacuole labeled by endosomal marker FM 4-64 (Xie et al., 2016). Given that ESCRT core complexes can be sequentially recruited and assembled in other organisms (Saksena et al., 2007; Otegui and Spitzer, 2008; Hurley, 2010; Henne et al., 2011) and that our yeast two-hybrid assay have identified the interactions between different subcomplexes in this study, the components of *F. graminearum* ESCRT-I, ESCRT-II, ESCRT-III should have similar localization pattern as FgVps27. To test this contention, each native ESCRT gene driven by native promoter was fused in-frame with the green fluorescent protein (GFP) and transformed into corresponding gene deletion mutant. After screening by PCR and GFP signal, positive transformants were obtained and the localization pattern was determined by confocal fluorescence microscopy. Similar to FgVps27, these GFP-ESCRT components were mainly found in the mobile punctate structures adjacent to the vacuolar membrane in conidia and mycelia (Figure 7 and Supplementary Figure 3 and Supplementary Videos S1–S6), which co-localized with the endocytic marker FM 4-64 labeling endosomes and vacuolar menbranes (Figure 7).

## Discussion

The ESCRT machinery consists of four core complexes, ESCRT-0, -I, -II, and -III and plays a critical role in capturing and sorting ubiquitinated proteins for lysosomal/vacuolar destruction (Katzmann et al., 2003; Tran et al., 2009). There has been a proliferation in studies of the order of recruitment and function of ESCRT machinery in yeast, mammalian and plants by using genetic and biochemical approaches over past two decades (Katzmann et al., 2001; Babst et al., 2002a,b; Bache et al., 2003; Lu et al., 2003; Cai et al., 2014; Gao et al., 2014). However, while homologs of most ESCRT components exist in plant fungal pathogens, little is known about the biological functions with the exception of ESCRT-0 in *F. graminearum*, which is critical for fungal development and virulence (Xie et al., 2016). To gain a better and comprehensive understanding of ESCRT machinery, we have systematically characterized the interaction network of ESCRT core complexes in *F. graminearum*, which has not been reported previously in filamentous fungi.

ESCRT-I contains four subunits including Vps23, Vps28, Vps37, and Mvb12 in yeast and humans (Richardson et al., 2011). However, only the Vps23 and Vps28 orthologs but not Vps37 and Mvb12 are found in *F. graminearum*, suggesting that other functionally equivalent proteins have replaced Vps37 and Mvb12 in *F. graminearum*. Otherwise, *F. graminearum* contains all other ESCRT orthologs (Richardson et al., 2011). To compare *F. graminearum* ESCRT interactome with those reported in other organisms, we have characterized interactions within *F. graminearum* ESCRT-I, -II and -III as well as between ESCRT-0 and ESCRT-I, ESCRT-I and ESCRT-II, and ESCRT-II and ESCRT-III. We have found that the two existing ESCRT-I components FgVps23 and FgVps28 can interact with each other and so are the ESCRT-II components. In *F. graminearum*, ESCRT-III contains four core subunits FgVps20, FgVps32, FgVps24, and FgVps2, which interact in that order. In addition, we find self-interaction of Vps32 in this study. These results are consistent with the previous data from yeast and humans (Martin-serrano et al., 2003; Von Schwedler et al., 2003; Bowers et al., 2004; Teis et al., 2008), indicating that interactions of the components within ESCRT -I, -II and -III are well-conserved across species. For interactions between the three ESCRT multi-protein complexes, the interactions of FgVps23-FgVps22, FgVps23-FgVps36, and FgVps28-FgVps36 link the ESCRT-I and ESCRT-II complexes while ESCRT-II components FgVps25 and FgVps36 interact directly with FgVps20, a protein in ESCRT-III complex, which connects the ESCRT-II and ESCRT-III complexes. Additional interactions including Vps28-Vps25 and Vps28-Vps22 (ESCRT-I and ESCRT-II), and Vps22-Vps20 (ESCRT-II and ESCRT-III) were previously identified in yeast (Bowers et al., 2004; Teis et al., 2008). Some new interactions between ESCRT-II and ESCRT-III including EAP30 (ortholog of *S. cerevisiae* Vps22)-Chmp6 (ortholog of *S. cerevisiae* Vps20), EAP30-Chmp4 (ortholog of *S. cerevisiae* Vps32), EAP20 (ortholog of *S. cerevisiae* Vps25)-Chmp4, EAP45 (ortholog of *S. cerevisiae* Vps36)-Chmp4 were also observed in mammals (Martin-serrano et al., 2003; Von Schwedler et al., 2003). Unlike *F. graminearum* and mammals, yeast *S. cerevisiae* appears not to exhibit interactions of Vps23-Vps22 and Vps23-Vps36 (ESCRT-I and ESCRT-II) (Martin-serrano et al., 2003; Von Schwedler et al., 2003; Bowers et al., 2004; Teis et al., 2008). Otherwise, the interactions between ESCRT multi-protein subcomplexes in *F. graminearum* are consistent with yeast and mammalian models for sequential recruitment and assembly of ESCRT complexes (Martin-serrano et al., 2003; Von Schwedler et al., 2003; Bowers et al., 2004; Teis et al., 2008, 2009; Raiborg and Stenmark, 2009; Adell and Teis, 2011). These results suggest that there may be some differences in the components of ESCRT complexes and interaction network between yeast, human and *F. graminearum*, but the basic machinery and interaction network seem to be conserved.

A previous study showed that FgVps27, the core component of the ESCRT-0 complex, was more sensitive to cell wall-damaging agents. In this study, all the ESCRT-I, -II, and -III gene deletion mutants are also hypersensitive to cell wall-damaging agents, suggesting defects in cell wall integrity. In addition, ESCRT components are critical for sexual reproduction. ESCRT mutants fail to produce any perithecium on self-cross plates. The mechanism of defects in response to cell wall stress and sexual reproduction is yet to be established but is likely related to endocytosis. In yeast, the block of cell wall stress sensor Wsc1p endocytosis resulted in defects in deposition of the cell wall and exhibited hypersensitivity to perturbation of cell wall synthesis (Piao et al., 2006). Moreover, Chvatchko and colleagues identified two mutants *end1* and *end2* defective in endocytosis and both of them also showed defect in mating pheromone response (Chvatchko et al., 1986). In this study, one of the marked defects in the ESCRT-I, -II, and -III gene deletion mutants is abnormal endocytosis. With time, FM4-64 can be internalized from the plasma membrane and transported into the vacuole in wild-type strain. However, this normal endocytic trafficking pathway is blocked in the mutants. Therefore, endocytic defects in ESCRT gene deletion mutants might account for the defects in response to environmental stimuli and sexual development.

In addition to ESCRT genes, many genes involved in endocytosis also have been reported to play critical roles in the development of different fungi. The deletion of SNARE protein Vam7 in *Magnaporthe oryzae* causes endocytosis defect and affects growth, sporulation and pathogenicity (Dou et al., 2011). Down-regulation of an endocytosis-associated gene *END4* in *Aspergillus oryzae* results in remarkable growth defect, alters hyphal morphology and exhibits higher salt and osmotic stress sensitivities (Higuchi et al., 2009). Yup1, an early endosomal t-SNARE protein, is important for functional endocytosis in *Ustilago maydis* (Wedlich-Söldner et al., 2000). Temperature-sensitive mutant strain *yup1*^*ts*^ fails to infect plants at higher temperature in contrast to the wild-type (Fuchs et al., 2006). These published studies, along with our results, suggest that proteins required for endocytosis are important for the development and virulence in various fungi.

The loss of ESCRT-I, -II, and -III components result in significant reduction in virulence, which is mainly due to severe growth defects. In addition, several factors may also contribute to the defects in pathogenesis. Previous studies showed that DON plays a critical role in fungal infection of plants as a virulence factor (Proctor et al., 1995; Desiardins et al., 1996) and in the current study, we have found that DON production and the expression of DON synthesis-related genes dramatically decrease in the ESCRT deletion mutants, possibly due to compromised endocytic trafficking and transport of these membrane-associated enzymes. Moreover, reduced hydrophobicity observed in the ESCRT deletion mutants may also contribute to the reduced pathogenicity on wheat head for the importance of cell surface hydrophobicity during plant infection (Kershaw and Talbot, 1998).

In summary, we have clarified the interactome of ESCRT machinery in *F. graminearum* by using yeast two-hybrid assay. In addition, we have also systematically characterized the biological function of ESCRT components in *F. graminearum* and found that the ESCRT mutants exhibit pleiotropic defects in growth, asexual and sexual reproduction, endocytosis, stress response, DON production and plant infection, demonstrating the importance of the ESCRT machinery in fungal development and virulence. To our knowledge, this is the first report for interactome and functional characterization of ESCRT machinery in filamentous fungi. Further identification of cargoes sorted by the ESCRT machinery should further clarify the ESCRT function and related networks in *F. graminearum*.

## Author Contributions

QX, AC, ZW, GL, and JZ conceived and designed the experiments. QX, AC, YZ, MY, and WX involved in the phenotype analysis of ESCRT deletion mutants. QX, AC, CZ, and WZ participated in the localization observation and DON detection of strains. QX and AC analyzed the data and wrote the manuscript. ZW, GL, and JZ revised the manuscript.

### Conflict of Interest Statement

The authors declare that the research was conducted in the absence of any commercial or financial relationships that could be construed as a potential conflict of interest.

## Supplementary Material

Supplementary Figure 1 Identification of orthologs of ESCRT components in *F. graminearum*. The protein sequences of ESCRT components from the budding yeast *S. cerevisiae* were used as queries.

Supplementary Figure 2 Deletion of the ESCRT component genes from *F. graminerum*. The gene replacement strategy was used to generate the targeted gene deletion mutants. In the schematic diagram of gene locus and gene replacement construct, all targeted genes were marked with white arrows, while black arrow indicated hygromycin phosphotransferase (*hph*) gene. Primers used for gene deletion and mutant identification were marked with small arrows. Fungal genomic DNAs were extracted and digested with restriction enzymes, subsequently subjected to southern blot analysis with probes. **(A)** Targeted gene deletion of *FgVPS23, Bam*H I digested DNAs revealed a 2.1 kb band in wild type PH-1 and 4.6 kb bands in mutants. **(B)** Targeted gene deletion of *FgVPS28, Sac* I digested DNAs revealed a 0.9 kb band in PH-1 and 3.4 kb bands in mutants. **(C)** Targeted gene deletion of *FgVPS22, Sac* I digested DNAs revealed a 0.6 kb band in PH-1 and 2.3 kb bands in mutants. **(D)** Targeted gene deletion of *FgVPS25, Xho* I digested DNAs revealed a 2.3 kb band in PH-1 and 2.9 kb bands in mutants. **(E)** Targeted gene deletion of *FgVPS36, Bgl* II digested DNAs revealed a 2.5 kb band in PH-1 and 5.8 kb bands in mutants. **(F)** Targeted gene deletion of *FgVPS20, Hin*d III digested DNAs revealed a 2.8 kb band in PH-1 and 3.5 kb bands in mutants.

Supplementary Figure 3 Localization of GFP–ESCRTs in vegetative mycelia. Shown are confocal fluorescent images indicating the localization of GFP–FgESCRTs in the punctate structures adjacent to the vacuolar in vegetative mycelia. Bars = 10 μm.

Supplementary Table 1 Yeast two hybrid performed in this study.

Supplementary Table 2 PCR primers used in this study.

Supplementary Video 1 Dynamics and mobility of FgVps23-GFP.

Supplementary Video 2 Dynamics and mobility of FgVps28-GFP.

Supplementary Video 3 Dynamics and mobility of FgVps22-GFP.

Supplementary Video 4 Dynamics and mobility of FgVps25-GFP.

Supplementary Video 5 Dynamics and mobility of FgVps36-GFP.

Supplementary Video 6 Dynamics and mobility of FgVps20-GFP.
