# Supplementary figures and images for: Component Interaction of ESCRT Complexes Is Essential for Endocytosis-Dependent Growth, Reproduction, DON Production and Full Virulence in Fusarium graminearum
Source: Front Microbiol. 2019 Feb 12;10:180. doi: 10.3389/fmicb.2019.00180 (PMC6379464; doi:10.3389/fmicb.2019.00180)

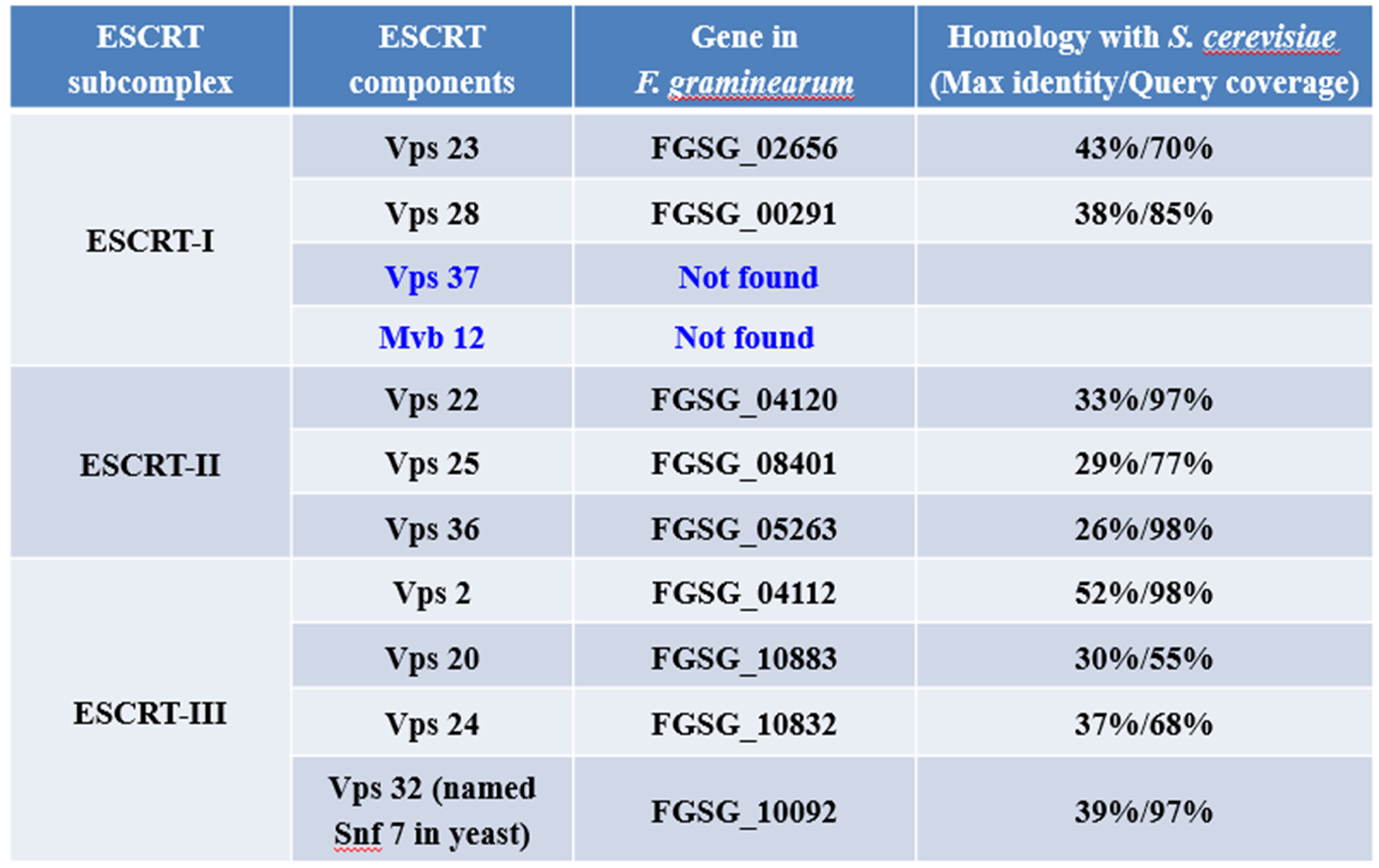

Supplement: Supplementary Figure 1 — Identification of orthologs of ESCRT components in F. graminearum. The protein sequences of ESCRT components from the budding yeast S. cerevisiae were used as queries. [file Image_1.TIF]

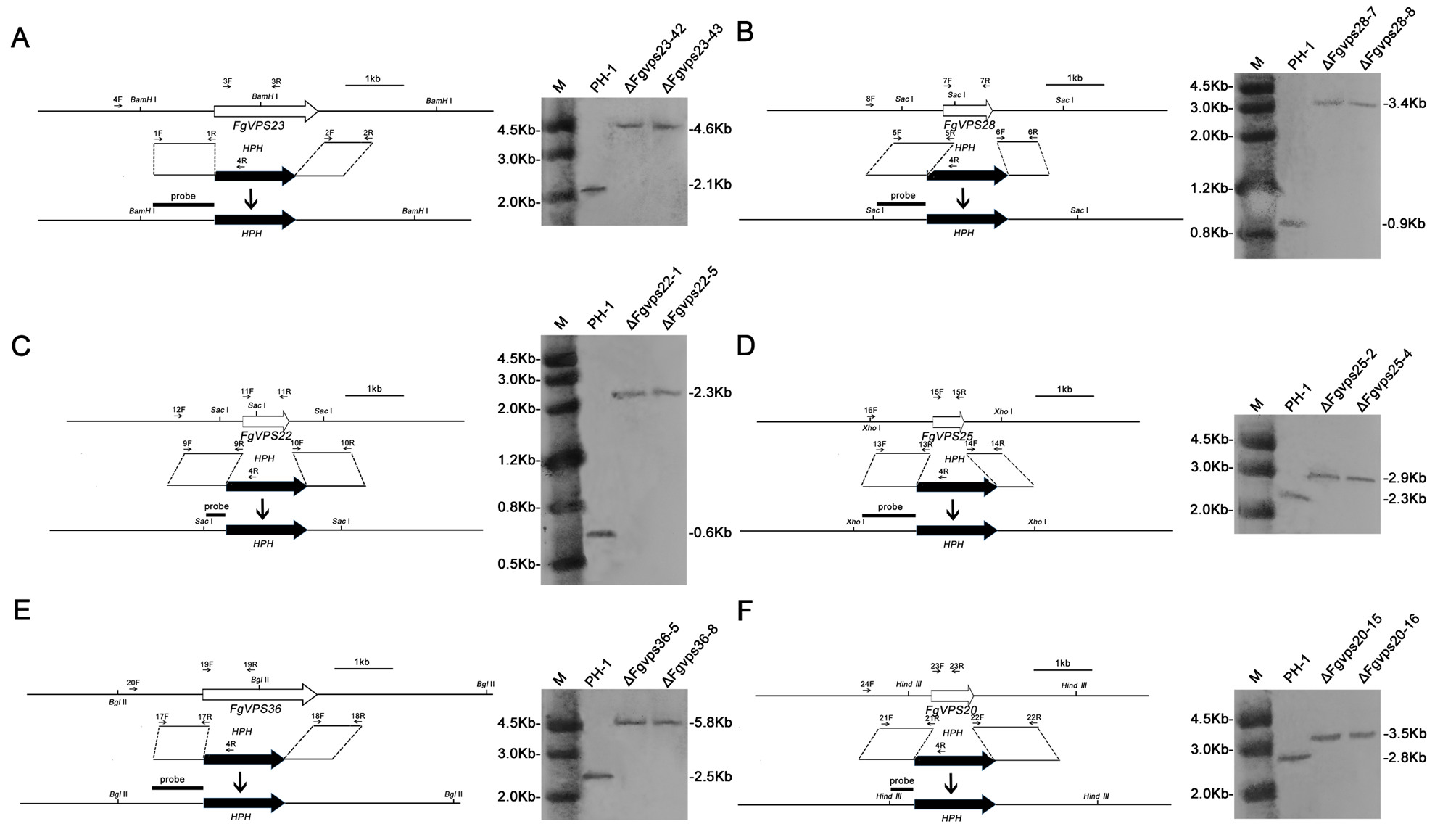

Supplement: Supplementary Figure 2 — Deletion of the ESCRT component genes from F. graminerum. The gene replacement strategy was used to generate the targeted gene deletion mutants. In the schematic diagram of gene locus and gene replacement construct, all targeted genes were marked with white arrows, while black arrow indicated hygromycin phosphotransferase (hph) gene. Primers used for gene deletion and mutant identification were marked with small arrows. Fungal genomic DNAs were extracted and digested with restriction enzymes, subsequently subjected to southern blot analysis with probes. (A) Targeted gene deletion of FgVPS23, BamH I digested DNAs revealed a 2.1 kb band in wild type PH-1 and 4.6 kb bands in mutants. (B) Targeted gene deletion of FgVPS28, Sac I digested DNAs revealed a 0.9 kb band in PH-1 and 3.4 kb bands in mutants. (C) Targeted gene deletion of FgVPS22, Sac I digested DNAs revealed a 0.6 kb band in PH-1 and 2.3 kb bands in mutants. (D) Targeted gene deletion of FgVPS25, Xho I digested DNAs revealed a 2.3 kb band in PH-1 and 2.9 kb bands in mutants. (E) Targeted gene deletion of FgVPS36, Bgl II digested DNAs revealed a 2.5 kb band in PH-1 and 5.8 kb bands in mutants. (F) Targeted gene deletion of FgVPS20, Hind III digested DNAs revealed a 2.8 kb band in PH-1 and 3.5 kb bands in mutants. [file Image_2.TIF]

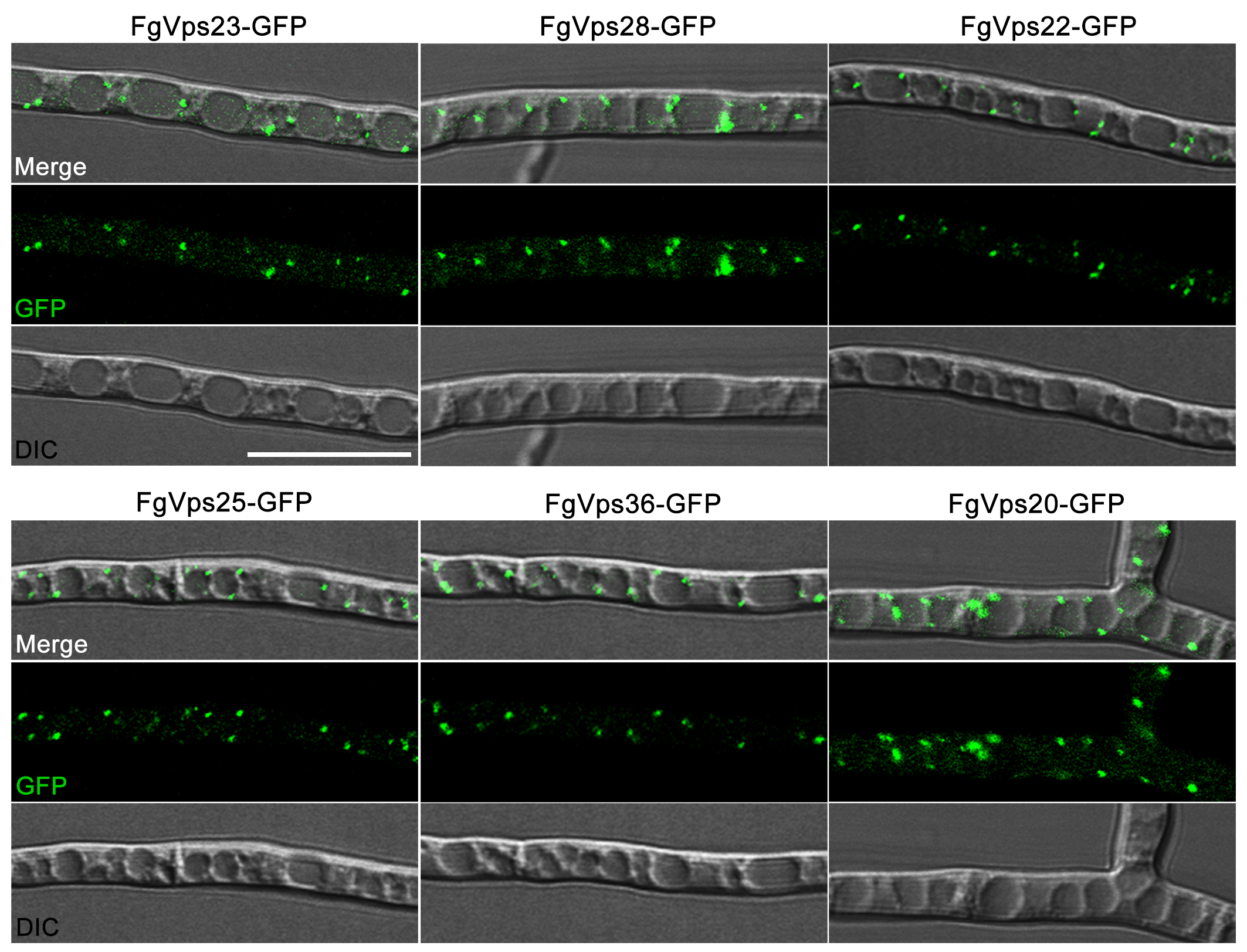

Supplement: Supplementary Figure 3 — Localization of GFP–ESCRTs in vegetative mycelia. Shown are confocal fluorescent images indicating the localization of GFP–FgESCRTs in the punctate structures adjacent to the vacuolar in vegetative mycelia. Bars = 10 μm. [file Image_3.TIF]
