# Supplementary material for: Component Interaction of ESCRT Complexes Is Essential for Endocytosis-Dependent Growth, Reproduction, DON Production and Full Virulence in Fusarium graminearum
Source: Front Microbiol. 2019 Feb 12;10:180. doi: 10.3389/fmicb.2019.00180 (PMC6379464; doi:10.3389/fmicb.2019.00180)
Supplement: Supplementary Table 1 — Yeast two hybrid performed in this study. [file Table_1.DOC]

**Supplementary Table 1. Yeast two hybrid performed in this study.**

| Prey-Bait Pairs | Interaction |
| --- | --- |
| FgVps23BD-FgVps27AD | Yes |
| FgVps23BD-FgVps28AD | Yes |
| FgVps22BD-FgVps25AD | Yes |
| FgVps22BD-FgVps36AD | Yes |
| FgVps25BD-FgVps36AD | Yes |
| FgVps23BD-FgVps22AD | Yes |
| FgVps23BD-FgVps25AD | No |
| FgVps23BD-FgVps36AD | Yes |
| FgVps22BD-FgVps28AD | No |
| FgVps25BD-FgVps28AD | No |
| FgVps36BD-FgVps28AD | Yes |
| FgVps20BD-FgVps32AD | Yes |
| FgVps20BD-FgVps24AD | No |
| FgVps20BD-FgVps2AD | No |
| FgVps32BD-FgVps32AD | Yes |
| FgVps32BD-FgVps24AD | Yes |
| FgVps32BD-FgVps2AD | No |
| FgVps24BD-FgVps2AD | Yes |
| FgVps36BD-FgVps20AD | Yes |
| FgVps36BD-FgVps32AD | No |
| FgVps36BD-FgVps24AD | No |
| FgVps36BD-FgVps2AD | No |
| FgVps22BD-FgVps20AD | No |
| FgVps22BD-FgVps32AD | No |
| FgVps22BD-FgVps24AD | No |
| FgVps22BD-FgVps2AD | No |
| FgVps25BD-FgVps20AD | Yes |
| FgVps25BD-FgVps32AD | No |
| FgVps25BD-FgVps24AD | No |
| FgVps25BD-FgVps2AD | No |

**Supplementary Table 2. PCR primers used in this study.**

| Primers | Names | Sequence(5'-3') | Application |
| --- | --- | --- | --- |
| 1F | FGSG_02656AF | CGGGCTTTCAGAGGTTTGG | FgVPS23  Deletion  (1F/R also used as probe of southern blot) |
| 1R | FGSG_02656AR | TTGACCTCCACTAGCTCCAGCCAAGCCACATACGCTGGTAAGGACGCT |
| 2F | FGSG_02656BF | GAATAGAGTAGATGCCGACCGCGGGTTTCTGACAGCTACGAGGTGAA |
| 2R | FGSG_02656BR | AAAGAAGCCGCAATGAAC |
| 3F | FGSG_02656OF | CGATGTGAACCGTGCCTAT | ΔFgvps23  mutant screen |
| 3R | FGSG_02656OR | GCTGCCATTGTTGTTGTGAT |
| 4F | H853 | GAGTTGAGATGTGGGTGGGC |
| 4R | FGSG_02656UA | ACATAAGAGGAATAATGGGAGC |
| 5F | FGSG_00291-AF | GCTCGTCCTCTTGTTCGT | FgVPS28  deletion |
| 5R | FGSG-00291-AR | TTGACCTCCACTAGCTCCAGCCAAGCCAGTGACTCTTGGAGATCGC |
| 6F | FGSG_00291-BF | GAATAGAGTAGATGCCGACCGCGGGTTCATGGCTATTAGAAGTGGTGTT |
| 6R | FGSG-00291-BR | GGAGGTTCAGGTCTGGGAT |
| 7F | FGSG_00291-OF | TTCATAATGATCCCACGACAA | ΔFgvps28  mutant screen |
| 7R | FGSG -00291-OR | AATCCCTGGTACGCCTGCT |
| 8F | FGSG_00291-UA | AGAGGAGGTAAGGGAGAATGGG |
| 9F | FGSG_04120-AF | ATGGGCGAAAACTCAGGC | FgVPS22  deletion |
| 9R | FGSG_04120-AR | TTGACCTCCACTAGCTCCAGCCAAGCCGGCGATTGTTGGAGAAGGA |
| 10F | FGSG_04120-BF | GAATAGAGTAGATGCCGACCGCGGGTTCCCGAGCGACCATGATA |
| 10R | FGSG_04120-BR | AAGTTCCTTCCCGACCAG |
| 11F | FGSG_04120-OF | TCGCAAAACTTCAACCTTC | ΔFgvps22  mutant screen |
| 12R | FGSG_04120-OR | TTTCTCAACCTCCCTCCTC |
| 13F | FGSG_04120-UA | TGAGGCTGAGGAGGTTGATGA |
| 14F | FGSG_08401-AF | GCCTGTGGCGTAACAAGCA | FgVPS25  Deletion  (14F/14R also used as probe of southern blot) |
| 14R | FGSG_08401-AR | TTGACCTCCACTAGCTCCAGCCAAGCCTGACGTGGGAGGGGTTCTAG |
| 15F | FGSG_08401-BF | GAATAGAGTAGATGCCGACCGCGGGTTCAAGTTCGGCACTGCTATC |
| 15R | FGSG_08401-BR | TCGTCCGCACTGTCAAAT |
| 16F | FGSG_08401-OF | TTCTTCACCCGCCAAACA | ΔFgvps25  mutant screen |
| 16R | FGSG_08401-OR | ACGCCTAATGAGTCGTCCTG |
| 17F | FGSG_08401-UA | CCCAGGTCTGCCGTGGTTAT |
| 18F | FGSG_05263-AF | ATTGAACCCTTGACGGAC | FgVPS36  Deletion  (18F/18R also used as probe of southern blot) |
| 18R | FGSG_05263-AR | TTGACCTCCACTAGCTCCAGCCAAGCCCAAACAACTTACCCCTCATAC |
| 19F | FGSG_05263-BF | GAATAGAGTAGATGCCGACCGCGGGTTTCACCAGGGCTTGAAATAATG |
| 19R | FGSG_05263-BR | ATGGGGATGGCGGAATAA |
| 20F | FGSG_05263-OF | TTGGCTTGTATGAGGGGTAA | ΔFgvps36  mutant screen |
| 20R | FGSG_05263-OR | CAGTTCTGAGTGTTTCGGATG |
| 21F | FGSG_05263-UA | ACTTGATTTAGGTAGGGGCTCG |
| 22F | FGSG_10883-AF | AGACTCAACTTTTTTCCCGT | FgVPS20  deletion |
| 22R | FGSG_10883-AR | TTGACCTCCACTAGCTCCAGCCAAGCCGACTTTACTCGCATTCCCA |
| 23F | FGSG_10883-BF | GAATAGAGTAGATGCCGACCGCGGGTTCGTTGGATACAGACCAGACA |
| 23R | FGSG_10883-BR | CGTGGGAAAGGAAGCAGA |
| 24F | FGSG_10883-OF | TTACATCTCTTCCTCCCCG | ΔFgvps20  mutant screen |
| 24R | FGSG_10883-OR | CTGCCGTTTCACCCATTA |
| 25F | FGSG_10883-UA | AAAGGCTTGCCGTCGTTG |
| 26F | 291 TF | AGGTCGTCTGCCTCTTCG | FgVPS28 probe |
| 26R | 291TR | CGGCTATTAGCTTGGTTATGAT |
| 27F | 4120TF | TCGAAGTATTAGGCACTGAATG | FgVPS22 probe |
| 27R | 4120TR | GGCGATTGTTGGAGAAGGA |
| 28F | 10883TF | GACTTAGGCTTGGGCTTATG | FgVPS20 probe |
| 28R | 10883TR | GACTTTACTCGCATTCCCAC |
| 29F | FGSG_02656KNTGF | AGGGAACAAAAGCTGGGTACCTCCAGGTGCTCTGAGATTTC | Construction  of GFP-FgVps23 fusion vector |
| 29R | FGSG_02656KNTGR | CAGGCATGCAAGCTTATCGATCGCAGCCAGGCCCATT |
| 30F | FGSG_00291KNTGF | AGGGAACAAAAGCTGGGTACCGCGTCGTTCTTGTTGTGTA | Construction  of GFP-FgVps28 fusion vector |
| 30R | FGSG_00291KNTGR | CAGGCATGCAAGCTTATCGATGGTCAAAGTCCTTCTAAATCC |
| 31F | FGSG_04120KNTGF | AGGGAACAAAAGCTGGGTACCTAACGAAGCGACGACTATT | Construction  of GFP-FgVps22 fusion vector |
| 31R | FGSG_04120KNTGR | CAGGCATGCAAGCTTATCGATACCTCCCTCCTCAGGATC |
| 32F | FGSG_08401KNTGF | AGGGAACAAAAGCTGGGTACCTGGGTAACTCTCGCCATTT | Construction  of GFP-FgVps25 fusion vector |
| 32R | FGSG_08401KNTGR | CAGGCATGCAAGCTTATCGATGTAGGACATCTGTGTCCATGC |
| 33F | FGSG_05263KNTGF | AGGGAACAAAAGCTGGGTACCCGGTCGGTTCGTCGTATT | Construction  of GFP-FgVps36  fusion vector |
| 33R | FGSG_05263KNTGR | CAGGCATGCAAGCTTATCGATTAAGAGGCCGCTTGCTTT |
| 34F | FGSG_10883 KNTGF | AGGGAACAAAAGCTGGGTACCGCCTGAAAGAAGAATACCCG | Construction  of GFP-FgVps20  fusion vector |
| 34R | FGSG_10883 KNTGR | CAGGCATGCAAGCTTATCGATTGCTGCAAGCATAGCTGG |
| 35F | TRI5QF | ACCCTCGGAGTGGGCTTATC | TRI5 qRT |
| 35R | TRI5QR | CTTCCAAACTCGCCTTCGG |
| 36F | TRI6QF | GTCGCTACTCAGAATGCCCTC | TRI6 qRT |
| 36R | TRI6QR | CCACCCTGCTAAAGACCCTC |
| 37F | TRI12QF | TTCCACAGTCATCTTTCCCCA | TRI12 qRT |
| 37R | TRI12QR | TCAAGTACGTCCTTATCCGCT |
| 38F | TUBLINQF | TATCCCATCATTACATCTACAGCCG | Tublin qRT |
| 38R | TUBLINQR | TGCCAGAAAGCAGCACCGA |
| 39F | FGSG_8545ADF | GGAATTCCATATG ATGATGAGTTGGTGGTCGTC | Yeast two hybrid AD-FgVps27  vector |
| 39R | FGSG_8545ADR | CCGGAATTC TTAAAACTCAATCAGGGCCTC |
| 40F | FGSG_2656BDF | TCAGAGGAGGACCTGCATATGATGCCCGTCCAGCAGCATGT | Yeast two hybrid BD-FgVps23 vector |
| 40R | FGSG_2656BDR | TCGACGGATCCCCGGGAATTCTCACGCAGCCAGGCCCATT |
| 41F | FGSG_291 BDF | TCAGAGGAGGACCTGCATATGATGATCCCACGACAAGGCT | Yeast two hybrid BD-FgVps28 vector |
| 41R | FGSG_291 BDR | TCGACGGATCCCCGGGAATTCTCAGGTCAAAGTCCTTCTAAATCC |
| 42F | FGSG_4120 BDF | TCAGAGGAGGACCTGCATATGATGTCTCGTAAAGGCGTAGG | Yeast two hybrid BD-FgVps22 vector |
| 42R | FGSG_4120 BDR | TCGACGGATCCCCGGGAATTCTCAACCTCCCTCCTCAGG |
| 43F | FGSG_8401 BDF | TCAGAGGAGGACCTGCATATGATGGCTGCTACCACCACC | Yeast two hybrid BD-FgVps25 vector |
| 43R | FGSG_8401 BDR | TCGACGGATCCCCGGGAATTCTTAGTAGGACATCTGTGTCCATG |
| 44F | FGSG_5263 BDF | TCAGAGGAGGACCTGCATATGATGTTTCTGAAACACATCGACC | Yeast two hybrid BD-FgVps36 vector |
| 44R | FGSG_5263 BDR | TCGACGGATCCCCGGGAATTCCTATAAGAGGCCGCTTGCTT |
| 45F | FGSG_10883 BDF | TCAGAGGAGGACCTGCATATGATGGGTGGGAATGCGAGTA | Yeast two hybrid BD-FgVps20 vector |
| 45R | FGSG_10883 BDR | TCGACGGATCCCCGGGAATTCTCATGCTGCAAGCATAGCTG |
| 46F | FGSG_10092 BDF | TCAGAGGAGGACCTGCATATGATGTGGGGTTGGTTCGGT | Yeast two hybrid BD-FgVps32 vector |
| 46R | FGSG_10092 BDR | TCGACGGATCCCCGGGAATTCTCACATAGCCATCTCGGCC |
| 47F | FGSG_10832 BDF | TCAGAGGAGGACCTGCATATGATGGAAACGTTTAAATCTCTCT | Yeast two hybrid BD-FgVps24 vector |
| 47R | FGSG_10832 BDR | TCGACGGATCCCCGGGAATTCCTAACTGCGAAGCGCCTCTA |
| 48F | FGSG_2656ADF | GTACCAGATTACGCTCATATGATGCCCGTCCAGCAGCATGT | Yeast two hybrid AD-FgVps23 vector |
| 48R | FGSG_2656ADR | ATGCCCACCCGGGTGGAATTCTCACGCAGCCAGGCCCATT |
| 49F | FGSG_291ADF | GTACCAGATTACGCTCATATGATGATCCCACGACAAGGCT | Yeast two hybrid AD-FgVps28 vector |
| 49R | FGSG_291 ADR | ATGCCCACCCGGGTGGAATTCTCAGGTCAAAGTCCTTCTAAATCC |
| 50F | FGSG_4120 ADF | GTACCAGATTACGCTCATATGATGTCTCGTAAAGGCGTAGG | Yeast two hybrid AD-FgVps22 vector |
| 50R | FGSG_4120 ADR | ATGCCCACCCGGGTGGAATTCTCAACCTCCCTCCTCAGG |
| 51F | FGSG_8401 ADF | GTACCAGATTACGCTCATATGATGGCTGCTACCACCACC | Yeast two hybrid AD-FgVps25 vector |
| 51R | FGSG_8401 ADR | ATGCCCACCCGGGTGGAATTCTTAGTAGGACATCTGTGTCCATG |
| 52F | FGSG_5263 ADF | GTACCAGATTACGCTCATATGATGTTTCTGAAACACATCGACC | Yeast two hybrid AD-FgVps36 vector |
| 52R | FGSG_5263 ADR | ATGCCCACCCGGGTGGAATTCCTATAAGAGGCCGCTTGCTT |
| 53F | FGSG_10883 ADF | GTACCAGATTACGCTCATATGATGGGTGGGAATGCGAGTA | Yeast two hybrid AD-FgVps20 vector |
| 53R | FGSG_10883 ADR | ATGCCCACCCGGGTGGAATTCTCATGCTGCAAGCATAGCTG |
| 54F | FGSG_10092 ADF | GTACCAGATTACGCTCATATGATGTGGGGTTGGTTCGGT | Yeast two hybrid AD-FgVps32 vector |
| 54R | FGSG_10092 ADR | ATGCCCACCCGGGTGGAATTCTCACATAGCCATCTCGGCC |
| 55F | FGSG_10832 ADF | GTACCAGATTACGCTCATATGATGGAAACGTTTAAATCTCTCT | Yeast two hybrid AD-FgVps24 vector |
| 55R | FGSG_10832 ADR | ATGCCCACCCGGGTGGAATTCCTAACTGCGAAGCGCCTCTA |
| 56F | FGSG_4112ADF | GTACCAGATTACGCTCATATGATGAATATCCTGGAATGGGC | Yeast two hybrid AD-FgVps2 vector |
| 56R | FGSG_4112 ADR | ATGCCCACCCGGGTGGAATTCCTATTTCCGCAGACTGTCC |
